# The Sexual Recidivism Rates of Women Are Still Low: An Updated Meta‐Analysis

**DOI:** 10.1002/cbm.70014

**Published:** 2025-10-13

**Authors:** R. Karl Hanson, Franca Cortoni, Jeffrey Sandler

**Affiliations:** ^1^ Department of Psychology Carleton University Ottawa Ontario Canada; ^2^ School of Criminology Université de Montréal Montreal Quebec Canada; ^3^ Research Foundation for Mental Hygiene New York New York USA

**Keywords:** base rates, female, meta‐analysis, prediction, sexual recidivism

## Abstract

**Background:**

Compared to men, women are less likely to sexual offend. Previous reviews found low rates of sexual recidivism among women. The last published meta‐analysis was based on studies from before 2010.

**Aims:**

Conduct an updated meta‐analysis of the sexual recidivism rates of women returned to the community. We expected the rates to be low and to decline the longer they remained sexual offence free in the community.

**Methods:**

Fourteen studies met selection criteria. Their publication/presentation dates ranged from 1998 to 2023. Results were presented as raw proportions as well as meta‐analytic averages.

**Results:**

Of the 4208 women, 3.1% (131) were known to have sexually reoffended. The rate was 2.4% during the first 5 years (64/2642, *k* = 8) and 1.1% between years 5 and 10 (6/535, *k* = 2). There was large and significant variability across studies (prediction intervals: < 0.001%–11%). The rates of violent recidivism (7.8%) and general (any) recidivism (30.1%) were substantially higher than the rate of sexual recidivism.

**Conclusions:**

This review confirms previous findings that the sexual recidivism rate of women is very low. Their risk is so low that it is unlikely to be reduced by sexual crime specific treatment or public protection measures (e.g., registration and notification). Instead, gender‐responsive interventions should focus on the women's risk for general criminal recidivism and strive to promote successful reintegration.

## 1 Introduction

Although there are many characteristics shared by men who commit crime and women who commit crime, there are also significant differences. One of the most salient differences is that women commit fewer crimes. Compared to men, women are less likely to be arrested or convicted for any crime, their crimes are less serious, they receive more lenient sentences and they are less likely to reoffend (Blanchette and Brown 2006). For example, 11% of US state prisoners are female (Durose and Antenangeli 2021). In England and Wales, women comprise 15% of arrests and 4% of the prison population (Ministry of Justice 2022). The violent recidivism rate of women is approximately half that of men (Durose and Antenangeli 2021).

Perhaps the biggest difference in criminal behaviour between men and women concerns sexual crimes. Women commit very few sexual offences, and, if they do, they are unlikely to reoffend. In a review of 17 studies from 12 countries, Cortoni et al. (2017) found that 2.2% of sexual offences reported to police were by women. Higher proportions of women (11.6%) are found in population‐wide victimisation studies, which include both officially reported and unreported offences. Although the prevalence rates vary based on the samples and research methods used, all studies find that men commit many more sexual offences than women. Nevertheless, a non‐negligible proportion of sexual offences are committed by women.

Previous studies have found that women who have committed sexual offences are less likely to sexually reoffend than men who have committed sexual offences. For example, in the large U.S. Department of Justice Study (Alper and Durose 2019), male prisoners with an index sexual offence had a sexual recidivism rate of 7.7% after 9 years. Among the 324 women with a history of sexual offending, their rate of sexual recidivism was too low to even report. The rate, however, is not zero. A solid estimate of the base rate of sexual recidivism among women would help advance gender‐responsive criminal justice policies and practices for this group (Cortoni 2024).

We are aware of two previous meta‐analytic reviews of the base rate of sexual recidivism among women with a history of sexual offending, both of which have sufficient limitations that justify an updated review. Cortoni et al. (2010) reported an overall sexual recidivism rate of 1.3% after approximately 6 years, which raised to 3.2% if one outlier (Vandiver 2007) was included. Of the nine sexual recidivism studies included in Cortoni et al. (2010), one had a very small sample size (*N* = 6; Hanson et al. 2007), another a short follow‐up period (2 years; Home Office 1998–2003) and two have been superseded by more recent versions (M. Wijkman et al. 2009; Vandiver 2007). Moreover, the studies mostly involved women released in the 1990s, which may or may not represent the reoffending patterns in more recent samples. The sexual crime rates in England and Wales have, for example, increased dramatically since 2013 (Office of National Statistics 2023), which raises the possibility of cohort changes in the characteristics and recidivism rates of women perpetrators of sexual crime.

In an unpublished doctoral dissertation, Moore (2020) reported an overall rate of 4.8% for sexual recidivism. Although Moore's meta‐analysis is relatively recent, it was limited to American samples (*k* = 8). Furthermore, it included two studies with questionable research designs (see next section; Epperson et al. 2016; D. M. Vandiver and Walker 2002), and another with incorrect recidivism calculations (Minnesota Department of Corrections 2007: 2 out of 41 is 4.8%, not the 9.8% indicated in tab. B of Moore (2020)). Consequently, there is a need for an updated meta‐analysis that (a) includes recent studies, (b) includes women from diverse countries (not just the US) and (c) meets high research standards.

### 1.1 What Qualifies as a Recidivism Study?

There are two statistics that inform the likelihood of recidivism: (a) the proportion of cases known to have reoffended more than once and (b) the proportion of a defined cohort who reoffend in the future. Although the distinction between these statistics is subtle, they answer different questions. Of the two, it is the later statistic about future reoffending that is most relevant for applied assessments. A straightforward method of estimating the likelihood of future recidivism is through prospective studies. In prospective studies, researchers have a principled method of identifying the index sexual offence that starts the time at‐risk. Then, after some follow‐up time, researchers identify which of these cases are known to have the outcome of interest. The method used to identify the index sexual offence will result in some cases already having had the outcome prior to the index offence. These cases are not *by this fact* classified as recidivists in prospective studies; instead, they are considered as cases having prior offences. Recidivism research studies typically find that cases with prior offences are more likely to commit future offences than cases who are before the courts for the first time.

In contrast, statistics concerning the proportion of cases who have offended on more than one occasion do not distinguish between prior offences and future offences. All cases with multiple offences are considered recidivists. This statistic quantifies the persistence of offending of the sample but does not inform the applied question of the likelihood of future offending. Furthermore, without a principled method of identifying the index sexual offence, there is no a priori way of distinguishing which of multiple occurrences is a prior offence and which is recidivism. If the researcher chooses the first occurrence as the starting point (index offence), all of the additional occurrences count as recidivism (and it is impossible to answer the question whether prior offences predict future offences). If the researcher chooses the last occurrence as the index offence, the observed recidivism rate would be zero.

The distinction between prospective and nonprospective recidivism studies is not absolute. A very common research design is to approximate a prospective study by identifying a cohort from a specific time and place (e.g., all women treated at Happy Valley Correctional Institute between 2010 and 2015), and then collect recidivism information sometime later (e.g., in 2022). Offences committed before 2010 are clearly prior offences, and those after 2015 are clearly recidivism. Researchers, however, must decide how to classify cases with more than one offence during the recruitment period (2010–2015). Selecting the first would maximise the recidivism rate; selecting the last would minimise the rate. Some researchers split the difference by randomly selecting one of multiple offences during the recruitment period as the index offence (e.g., Helmus et al. 2021).

### 1.2 The Current Study

The aim of the current meta‐analysis was to estimate the likelihood that women with a sexual offence history would be found to have reoffended with a subsequent sexual offence after they returned to the community. Although fully prospective studies provide the best evidence, we accepted recidivism studies that closely approximated prospective designs. We summarised the rates of violent and general (any) recidivism, if reported, for studies that also included sexual recidivism. As in previous reviews (Cortoni et al. 2010; Moore 2020), we expected the violent and general recidivism rates to be substantially higher than the rate of sexual recidivism.

Another goal was to consider the extent to which the risk of sexual recidivism declines the longer the women remained sexual offence‐free in the community. Most recidivism (of all types) occurs shortly after release (Blumstein and Nakamura 2009; Bushway et al. 2022). Among men who have committed a sexual offence, their risk also predictably declines the longer they remain at‐risk without sexually reoffending (Hanson et al. 2018; Thornton et al. 2021). On average, their expected sexual recidivism risk is cut in half for every 5 years they remain sexual offence‐free (Hanson et al. 2014). For example, if their initial risk was 20% after 5 years, it would decline to 10% for years 5 to 10, and to 5% for years 10–15. We expected the same pattern to apply to the sexual recidivism risk of women.

## 2 Method

### 2.1 Inclusion and Exclusion Criteria

Studies were included if they reported the sexual recidivism rate of at least 10 women after an average follow‐up period of at least 3 years in the community. The initial sample, as well as the recidivism information, had to be based on criminal justice involvement (arrest, charge, or conviction). Qualifying offences were sexually motivated offences against an identifiable victim, child sexual exploitation offences (CSEM, e.g., possessing images depicting adult‐child sex), and offences related to the prostitution of children. We excluded offences related to sex trade work among adults. Sexual recidivism included a charge, conviction, or reincarceration for a new sexual offence. Violent recidivism was defined as a charge, conviction or incarceration for a new violent offence (including sexual offences). Any recidivism was defined as any new charge, conviction or incarceration for any type of crime (sexual, violent, nonviolent). Consequently, the categories of recidivism are cumulative rather than mutually exclusive.

We included fully and partially prospective studies. To qualify as a prospective study, the study must have a clearly defined follow‐up period and a principled method of identifying the index sexual offence that was independent of the number of offences in the recruitment and follow‐up periods. We excluded studies, for example, that only estimated the proportion of cases with more than one occurrence during the recruitment period, or that only reported the proportion of cohort who had a prior sexual offence. Further description of the sample selection criteria is available in the coding manual (see Supporting Information S1).

### 2.2 Search Strategy

**FIGURE 1:**
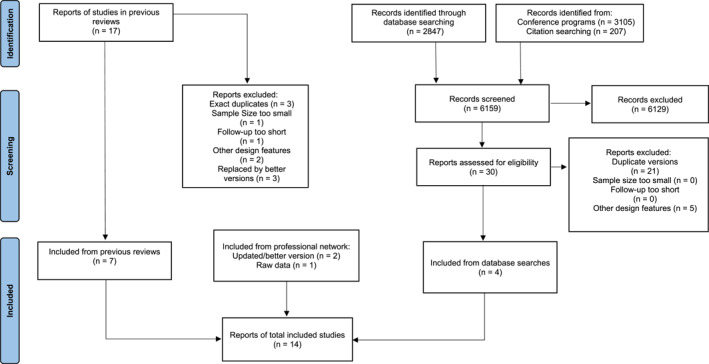
Search activities and results.

Seven unique studies were retained from previous reviews. Four of the nine studies in Cortoni et al. (2010) were retained: two were excluded based on design (Hanson et al. 2007; Home Office Statistics [1998–2003]) and three were replaced with more recent (or more complete) versions (D. M. Vandiver et al. 2019; Wijkman and Bijleveld 2013; Williams et al. 1998). Six of the eight studies in Moore (2020) were retained: two were excluded based on design (Epperson et al. 2016; D. M. Vandiver and Walker 2002). Data base searches identified four additional studies. Preliminary database searches were conducted in early 2023, and then updated in February/March, 2024, and again in October, 2024 (see Figure 1). The following databases were searched: Google Scholar, PsycINFO, EBSCOhost Web, ProQuest, and the conference programs of three relevant professional organisations. The primary search terms were variations on the following: “women,” “sex offenders,” “female,” “sexual offence,” “sexual offending,” “sexual recidivism,” “recidivism” and “re‐offending.” The exact search terms and sources are described in Supporting Information S2. The professional networks of the authors identified one additional data set (Sandler 2023) as well as updated (or more complete) versions of two studies (Wijkman and Bijleveld 2013; Williams et al. 1998). Three authors of the included studies provided additional data that was used in this meta‐analysis. The final tally included 14 studies with the total sample size of 4208 women.

### 2.3 Procedure

Each study was coded independently by two coders using a 12‐page coding manual (see Supporting Information S1) with the final coding based on consensus. Rater reliability was not assessed.

### 2.4 Aggregation of Findings

Meta‐analytic averages were computed using the arcsin variance stabilisation transformation (*Ă*), defined as $Ă=2\;\arcsin\sqrt{p}$ (Hanson 2022; Fleiss et al. 2003). For studies in which there were no recidivists, the recidivism rate (*p*) was estimated as 1/4*n* (i.e., Bartlett's adjustment, see Eisenhart 1947, § 4.3; Cohen 1988, 183). Bartlett's adjustment is a form of continuity correction that involves replacing zero with 0.25, which allows the analyses of empty cells and improves the concordance of the data to the distributional assumptions of the statistical models.

Aggregation of the effect sizes (*Ă*) was conducted using both fixed‐effect and random‐effects meta‐analyses (Borenstein et al. 2021), using the *metafor* package for R version 4.2.2 (R Core Team 2023; Viechtbauer 2010). Variability across studies was indexed by Cochran's *Q*, *I* ^2^ and prediction intervals. Cochran's *Q* is a significance test of whether the observed variability across studies is more than expected by chance. Based on *Q*, *I* ^2^ is an effect size indicator of the amount of heterogeneity across studies (Borenstein et al. 2021). *I* ^2^ values of 25%, 50% and 75% are considered low, moderate and high, respectively (Borenstein et al. 2021). Prediction intervals indicate the range of values expected should new studies be conducted on the same topic (Borenstein et al. 2021).

Outliers were identified with the *leave1out* analysis in the *metafor* package, using the decision rules proposed by Hanson (2022, 265–266). If the effect size was the highest or lowest and removing it reduced the *Q* by 50%, the effect was considered an outlier and removed from the main analyses. If *Q* was still significant after an outlier was removed, the remaining effect sizes were checked for outliers, and removed if identified.

In response to editorial feedback, post hoc analyses were conducted that examined the relationship between the overall sexual recidivism rate and (a) the average follow‐up time per study and (b) the length of the recruitment period (the difference in years between when the first and the last woman entered the cohort). These meta‐regressions were conducted using both fixed‐effect and random‐effects models. Readers should be aware that fixed‐effect models are expected to be too liberal (finds patterns that are not there) whereas the random‐effects models are too conservative (fails to detect patterns that are there; Overton 1998).

The meta‐analyses were run independently by two data analysts, with minor discrepancies resolved by consensus (mostly rounding errors).

## 3 Results

### 3.1 Characteristics of Studies

Eleven of the 14 studies were from the United States, with one each from Australia, Canada and the Netherlands. Sample size ranged from 41 to 1466 (median of 140; Total *N* = 4208). Six studies were published in peer reviewed journals; the others were conference presentations (3), government reports (2), doctoral dissertations (2) or raw data (1). Half were framed as studies of the sexual recidivism rate of women; in the others, this information was incidental to a larger purpose (e.g., validating a risk tool). Date of presentation/publication ranged from 1998 to 2023 (median of 2013). The start of the at‐risk period ranged from 1984 to 2007 (median of 1993), with the end of follow‐up ranging from 1994 to 2021 (median of 2011). The average follow‐up period ranged from 48 to 226 months (4–19 years), with a median of 94 months (8 years). The length of the recruitment period for identifying the cohort ranged from 10 to 20 years (median of 14 years, *k* = 11). For six of the nine studies that reported the dates, the end of the follow‐up period was < 1 year after the end of the recruitment period. Consequently, most of the recidivism events occurred during the recruitment period for the cohort.

Most of the samples were released from institutions (5) or were combined samples that included women who received institutional or community sentences (7). Most samples (10) were routine/complete (i.e., not preselected on risk or treatment needs). The extent to which the women received sexual crime specific treatment was largely unknown (7); none of the samples were identified as predominantly treated. The median age of the samples was 32 years (range from 28 to 39). One sample included a small proportion of female persons under the age of 18. All samples were predominantly White (58%–93%). Offences involving the prostitution of children were included in six of the eight studies that provided this information. The proportion of child prostitution cases was small in these studies (1.8%–5.0%) and could not be disaggregated from the overall sample.

The follow‐up period was either variable (7), fixed (1) or both fixed and variable (6). Seven studies used only one source of recidivism information (typically state [US] or national records [Canada, Netherlands]). One study used four sources, and the remainder used either 3 sources (2), 2 sources (2) or did not specify (2). The most common recidivism criterion was arrest (7) or conviction (5). Six studies indicated that they adjusted follow‐up time for periods in prison or a secure hospital (street time). Four studies indicated that they excluded pseudo‐recidivism (new charges/convictions for behaviour that predated the start of the at‐risk period). Only four studies reported attrition during follow‐up, which was generally small (0%, 0%, 2%, and 15%).

### 3.2 Recidivism Rates

In the total sample of 4208 women, 3.1% (131) were known to have sexually reoffended. The sexual recidivism rate was 2.4% during the first 5 years (64/2642, *k* = 8) and 1.1% between years 5 and 10 (6/535, *k* = 2; see Table 1). The rate of new violent charges/convictions was 7.8% (265/3414, *k* = 10, median follow‐up of 94 months). The rate of any recidivism was 30.1% (1091/3625, *k* = 10, median follow‐up of 87 months).

**TABLE 1:** Summary of sexual recidivism studies for women.

Source	Country	Recidivism type	Mean follow‐up (years)	Sexual recidivism rate % (*n* recidivists/total)		
				Overall	Within 5 years	Between 5 and 10 years
Bader et al. (2010)	U.S.—Nebraska	Charges	4.9	17.5 (10/57)	—	—
Broadhurst and Loh (2003)	Western Australia	Arrest	5.7	0 (0/43)	0 (0/21)	
Duwe et al. (2023)	U.S.—11 states	Conviction	4.0	3.2 (14/442)		
Marshall et al. (2021) McCoy (2015)	U.S.—Texas (2000–2014)	Arrest	7.0	3.5 (26/739)	3.8 (20/526)	—
McGinnis (2015)	U.S.—Iowa	Conviction	8	1.9 (2/105)	—	—
Minnesota Department of Corrections (2007)	U.S.—Minnesota	Arrest	8.4	4.9 (2/41)	—	—
Mowrer (2013)	U.S.—Oregon	Convictions	6.4	6.7 (4/60)	—	—
Peterson et al. (2001)	U.S.—Kentucky	Conviction	5.5	0 (0/113)	0 (0/56)	—
Sandler (2023)	U.S.—New York State	Arrest	8.0	0.60 (1/167)	0.88 (1/114)	
Sandler and Freeman (2009)	U.S.—New York State	Arrest	≈ 10	2.2 (32/1466)	1.8 (19/1041)	—
Tatar and Straveler (2015)	U.S.—Wisconsin (1992–2010)		≈ 9	0.55 (1/182)	0.65 (1/154)	0 (0/87)
D. M. Vandiver et al. (2019)	U.S.—Texas (1988–2001)	Arrest	18.8	7.2 (34/471)	4.5 (21/469)	1.3 (6/448)
Wijkman and Bijleveld (2013)	Netherlands	Conviction	13.2	1.1 (3/261)	0.77 (2/261)	—
Williams et al. (1998)	Canada	Charges	7.6	3.3 (2/61)	—	—
	Total (*n*/*N*)			3.1 (131/4208)	2.4 (64/2642)	1.1 (6/535)

**FIGURE 2:**
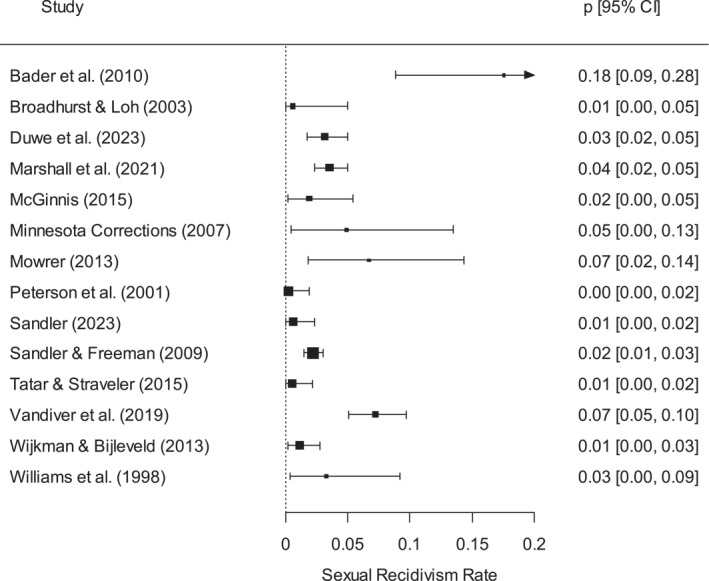
Forest plot of observed sexual recidivism rates for women.

Meta‐analysis indicated large and significant variability across the 14 studies (*Q* = 66.8, *p* < 0.001; *I* ^2^ = 80.5; see Figure 2 and Table 2). Both the fixed‐effect and random‐effects analyses resulted in an overall average of 2.8% (95% confidence interval [CI] of 2.3%–3.3% for fixed‐effect; 1.4%–4.6% for random‐effects). The prediction interval ranged from near zero to 11%, which was another indicator of large between‐study variability. No study was identified as a statistical outlier.

**TABLE 2:** Meta‐analytic summary of the sexual recidivism rates of women.

Number of studies (sample size)		*Q* (*p*)	*I* ^2^	Meta‐analytic averages (%) with 95% confidence intervals		
				Overall	First 5 years (0–5 years)	Next 5 years (5–10 years)
14 (4208)	Fixed‐effect	66.8 (< 0.001)	80.5	2.8 (2.3, 3.3)		
	Random‐effects			2.8 (1.4, 4.6)		
	Prediction interval			< 0.001, 10.7		
8 (2670)	Fixed‐effect	23.0 (0.0017)	69.6		2.2 (1.7, 2.8)	
	Random‐effects				1.8 (0.84, 3.1)	
	Prediction interval				< 0.001, 5.6	
2 (602)	Fixed‐effect	1.1 (0.29)	11.8			1.1 (0.40, 2.2)
	Random‐effects					1.1 (0.29, 2.3)
	Prediction interval					0.21, 2.6

Among the eight studies that reported sexual recidivism using a fixed 5‐year follow‐up period (*n* = 2670), the meta‐analytic average was 2.2% (1.7%–2.8%) using the fixed‐effect model and 1.8% (0.8%–3.1%) using the random‐effects model. The prediction interval ranged from essentially zero to 5.6%. There was significant and moderate variability in the 5‐year data; no study was identified as a statistical outlier. Among the two studies that reported recidivism rates for years 5–10, both fixed‐effect and random‐effects models resulted in an average rate of 1.1% (95% CI of 0.40%–2.2% for fixed‐effect; 0.29%–2.3% for random‐effects). The prediction interval ranged from 0.21% to 2.6%. Both studies provided similar results (nonsignificant *Q*, small *I* ^2^, and narrow prediction intervals; see Table 2).

The length of the follow‐up period (in years) was significantly and *positively* related to the overall sexual recidivism rate (in *Ă* metric) in the fixed‐effect meta‐regression (*β* = 0.00914, 95% C.I. of 0.00178–0.0165) but not in the random‐effects meta‐regression (*β* = 0.00547, 95% C.I. of −0.0234 to 0.0343). The length of the recruitment period was significantly and *negatively* related to the overall sexual recidivism rates in the fixed‐effect meta‐regression (*β* = −0.0198, 95% C.I. of −0.0282 to −0.0114) but not in the random‐effects meta‐regression (*β* = −0.0279, 95% C.I. of −0.0583 to 0.00253; *k* = 11). The same pattern held without Bader et al. (2010), which had the highest recidivism rate and tied for the shortest recruitment period (10 years).

## 4 Discussion

This review confirmed previous findings that the sexual recidivism rates of women are low. The overall rate was about 3%. When the follow‐up time was restricted to 5 years, the rate was 2%. The sexual recidivism rate between years 5 and 10 was 1%. Although only two studies reported the rates between years 5 and 10, the pattern exactly matches the pattern observed for men (i.e., risk is cut in half for every 5 years sexual offence free in the community; Hanson et al. 2014).

There was, however, significant variability across the studies, with rates ranging from zero (Broadhurst and Loh 2003; Peterson et al. 2001) to 17.5% (Bader et al. 2010). The reasons for this variability are not known. We did not plan meta‐regressions using moderator variables because there were too few studies for stable results (post hoc analyses are discussed below). However, the study by Bader et al. (2010) is noteworthy because of the particular attention they accorded to identifying sexual recidivism events in unofficial sources (e.g., child protection records). That unofficial records would produce higher estimates (28.1%) than official records (17.5%) is not surprising; what is surprising is the high rate Bader et al. (2010) found based on official records (17.5%). Given that their sample size was modest (57) and was not identified as a statistical outlier, it could be just a chance finding that awaits replication.

Within the criminal justice system, the principle goal of sexual offender treatment programs is to help the person not sexually reoffend. When the risk of committing a new sexual offence is very low, the utility of such specialised interventions becomes questionable in terms of improved outcomes. If women with a sexual offending history reoffend, it is most likely to be with a nonsexual offence. As such, their risk for sexual recidivism is already so low that they are unlikely to benefit from sexual crime specific interventions. This is not to say that women convicted of sexual offences would not benefit from some forms of interventions to improve their community functioning and reduce their risk of overall reinvolvement with the criminal justice system. In this set of studies, the general (any) recidivism rate was 30%, which is similar to the rates of other samples of women in the criminal justice system. Consequently, we recommend that women with a sexual offence history be offered gender responsive interventions that consider, as needed, the interrelated levels of trauma, mental health issues, substance abuse and other criminogenic problems (e.g., offence‐supportive cognitions) typically present in justice‐involved women (Van Voorhis 2022). Only in truly exceptional cases would specialised treatment for sexual offending behaviour be required.

The current review only examined the overall rates in aggregated samples; consequently, it is possible that some individual women may present a clinically significant risk for sexual reoffending. The ability to identify such women would be helpful to practitioners guided by the Risk‐Need‐Responsivity model of correctional rehabilitation (Andrews et al. 1990; Bonta and Andrews 2024). This is difficult. Static‐99R, the most commonly used risk tool for men (Helmus et al. 2022), does not work for women (Marshall et al. 2021). McGinnis (2015) found that the Iowa Sex Offender Risk Assessment (ISORA) was positively associated sexual recidivism for women, but the study included only two women who reoffended sexually (out of 105). Duwe et al. (2023) found that his newly created risk tool (Adult Dynamic Validated Instrument for Sex Offence Recidivism; ADVISOR) worked equally well for men and women. The study, however, included only 14 women who had reoffended sexually (out of 442; recidivism rate = 3.2%). One unusual feature of the Duwe et al.’s study was that the overall rate of sexual recidivism was higher for women (3.2%) than for men (2.5%). This raises questions about how sexual recidivism was measured in the 11 different states that contributed data to this project. Although Duwe et al. (2023) used appropriate statistical methods and took care to avoid overfitting their data, prudent evaluators may wish to wait for further research before using the ADVISOR with women.

In the absence of a validated risk tool to assess risk of sexual recidivism, one plausible approach to risk assessment is to ground estimates in the base rates, and then adjust up or down based on risk and protective factors (Kahneman et al. 2021). The current study provides a solid estimate of the base rate. Less is known about risk and protective factors. There is some evidence for the following risk factors: (a) a history of prior sexual offences (Marshall et al. 2021), (b) any prior convictions, particularly if involving a child victim (Sandler and Freeman 2009), (c) any noncontact sexual offences (Marshall et al. 2021) and (d) being a solo offender against a (young) male victim (Cortoni et al. 2015; S. M. Williams and Nicholaichuk 2001). The presence of any of these factors would suggest an elevated risk compared to the typical female charged/convicted of a sexual offense. How much these factors would increase the risk is not known.

The overall rate of sexual recidivism among women is lower than it is among men. Compared to the 3% rate in this meta‐analysis, meta‐analyses of male samples typically find sexual recidivism rates between 5% and 15% (Lee and Hanson 2021; Lussier et al. 2023). One limitation when comparing these meta‐analyses is that the men and women were drawn from different samples at different times; nevertheless, the same pattern holds when the men and women are matched on location, follow‐up period, and recidivism criteria. Of the samples included in the current meta‐analysis that directly compared the rates for men and women, Broadhurst and Loh (2003) observed a 33% sexual recidivism rate for men compared to *no* sexual recidivism for women. In Minnesota Department of Corrections (2007), the rate for men was 11.8% compared to 4.2% for the women. In New York, the rate was 5.4% for men compared to 2.2% for women (Freeman and Sandler 2008). In Wisconsin, the rate was 3.5% for men compared to 0.5% for women. The sole exception to this pattern was the study by Duwe et al. (2023) in which the sexual recidivism rate for women (3.1%) was higher than that for men (2.5%).

The low rates of sexual recidivism of women questions the utility of public protection measures being applied to women with a history of sexual offending. Many countries attempt to manage the risk of sexual offenders through civil (noncriminal) measures, such as registration, community notification and residence restrictions. Although the effectiveness of such measures has not been established (Savage and Windsor 2018; Zgoba and Mitchell 2021), if they are to work at all, the persons subject to these measures should be higher risk for sexual offending than other persons in the general population. For women, this does not appear to be the case. Their sexual recidivism rates are in the same range as that of persons with nonsexual convictions (2% after 5 years; Kahn et al. 2017) and men in the general population (2% lifetime incidence; Lee et al. 2023).

### 4.1 Limitations

Few of the studies used data that was originally collected for the purpose of assessing the likelihood of sexual recidivism among women. Given the low base rates, researchers rarely had the patience for true prospective designs. Instead, most of the studies involved secondary analysis of existing data, which may be weakly aligned with the question of future recidivism. There was little attention paid to pseudo‐recidivism (new charges for crimes committed prior to the index offence release date) or to attrition. Most researchers seemed to assume that their data were complete. This is unlikely to be the case. Selective attrition of low risk cases (nonrecidivists) is common in retrospective analysis of decades‐old administrative data (Hanson and Nicholaichuk 2000). There was also little attention paid to differencing women convicted of contact or CSEM offences from those convicted of promoting prostitution of a minor. However, this latter group presents with very different offending patterns and histories, and different recidivism rates, compared to the first group (Cortoni et al. 2015; Sandler and Freeman 2009).

Most studies had recruitment periods that overlapped with their follow‐up periods, which could have forced researchers to arbitrarily choose the index offence for women with more than one offence during the recruitment period. Although all studies used a principled method of identifying the index offence, coding decisions of the authors of the original studies may have introduced bias. To check for possible effects of long recruitment periods, we conducted post hoc meta‐regressions with the length of the follow‐up time and recruitment periods as predictors. As expected, longer follow‐up times were associated with high recidivism rates (in the fixed‐effect analysis). Contrary to expectation, the length of the recruitment period was associated with *low* recidivism rates (again, in the fixed‐effect analysis only). One possible explanation is that short recruitment periods may concentrate cases that are higher risk than average (Rhodes et al. 2016). The reason for this concentration is that low risk cases quickly exit the criminal justice system whereas higher risk cases return again and again to be counted in future cohorts. Consequently, studies with short recruitment periods are packed with higher risk cases because such cases are almost always there. It is not clear, however, that this explanation applies to the current finding because even the shortest recruitment periods were quite long (10 years).

Another limitation is that the current study relied on officially recorded sexual offences, both to identify the women at‐risk and to measure recidivism. Many sexual offences are never reported to police, and not all reports result in officially recorded charges and convictions. Although the extent of underestimation remains a topic of debate in the professional community, it is likely that the dark figure of undetected sexual offences is large. Whether it is larger for women than for men is not known. Cortoni et al. (2017) found that women comprised 2.2% of the sexual offence perpetrators in police statistics whereas they comprised 11.6% in community victimisation surveys, suggesting that relatively fewer sexual offences by women are reported to police. Consequently, it is difficult to generalise from the very low observed sexual recidivism rate of women to a reliable reoffending rate estimate that includes undetected offences. Nevertheless, the data in this review provides strong evidence that very few women with a known history of sexual offending go on to commit any new sexual offences of the type that victims decide to report, and that the police and courts decide are worthy of criminal justice intervention, with the higher likelihood of the latter for cases where the woman already has a known history of sexual offending.

## 5 Conclusions

Sexual crime is inherently shaped by cultural and social forces. All modern societies define permitted and prohibited sexual behaviours, as well as which prohibited behaviours are worthy of criminal justice intervention. These boundaries, however, are not static (consider the #MeToo Movement). Consequently, findings concerning sexual offending merit periodic updates to ensure their continued accuracy. This is the first comprehensive review since 2010 of the sexual recidivism rates of women. We found that little has changed: the observed sexual recidivism rates remain low (around 3%). They are so low as to raise questions about the utility of including women in broad public protection measures such as sexual offender registration and notification. Although these women are likely to have criminogenic needs worthy of intervention, narrowly focussed sexual crime specific treatment cannot be expected to reduce sexual recidivism when the base rate is already so low. Instead, we recommend that women with a history of sexual offending be offered gender responsive interventions that consider the full suite of problems typically present in justice‐involved women (Cortoni 2024).

## Ethics Statement

Ethics approval was not sought because we used only publicly available data.

## Conflicts of Interest

The authors declare no conflicts of interest.

## Supporting information


Supporting Information S1

Supporting Information S2

## Data Availability

Data sharing is not applicable to this article as no new data were created or analyzed in this study.
