# Supplementary material for: The Sexual Recidivism Rates of Women Are Still Low: An Updated Meta‐Analysis
Source: Crim Behav Ment Health. 2025 Oct 13;36(2):53–62. doi: 10.1002/cbm.70014 (PMC13063210; doi:10.1002/cbm.70014)
Supplement: Supplementary file 1 — Supporting Information S1 [file CBM-36-53-s002.pdf]

CODING MANUAL

Used in

The Sexual Recidivism Rates of Women are Still Low

RK Hanson, F Cortoni, and J Sandler

Version November 11, 2024

Study Purpose:

To estimate the likelihood of sexual recidivism among women with a history of sexual offending. This is an update to Cortoni et al. (2010). Although sexual recidivism is the focus of this meta-analysis, it will also address violent recidivism, and any criminal recidivism.

Inclusion Criteria:

1. Women with a history of sexual offending as documented by criminal justice involvement (arrested, charged, or convicted).
    a. Include contact and non-contact offences (Static-99R Category A offences)
    b. Include sexual exploitation of children (e.g., prostitution of children)
    c. Include possession/transmission of sexual exploitation materials
    d. Include sexually motivated offences, even if name is nonsexual (e.g., kidnapping, corruption of a minor)
2. The sample must be predominantly adults (18 + at time of index offence). Samples may include up to 10% of participants who were less than 18 at the time of the index sexual offence behaviour.
3. No nationality or language restrictions. That being said, the team has language proficiency only in English, French (FC, RKH), and Italian (FC); searches will only be conducted in these languages. If relevant articles in other languages are identified, however, we will seek assistance with translation and coding (e.g., calling in favours from bilingual colleagues).
4. Recidivism information. To be included the study must report the sexual, violent, or general (any) recidivism rates after a follow-up period. Studies that simply report the proportion of women who have more than one offence are excluded.
    a. The study should specify the time period used to identify the cohort (e.g., all women released between July 15, 2003 and August 1, 2010).
    b. The period of time used to identify the cohort must be different from the follow-up time. Individuals who have more than sexual offence within the cohort selection period are coded as having a prior sexual offence, and not to be sexual recidivists
    c. The average follow-up period must be at least three years (36 months).
    d. Official recidivism as indicated by arrest, charge, conviction or reincarceration for criminal behaviour.
5. Studies could include peer reviewed publications, book chapters, conference presentations, government reports, officially recidivism statistics drawn from websites or through direct communication with government agencies, and unpublished data obtained directly from the researchers.

6. No year/time restrictions. However, given that Cortoni et al. (2010) conducted a thorough search of studies prior to 2010, the search focussed on studies produced in 2008 or later.

Exclusion Criteria:

1. Sample is mixed-gender or male-only.
2. Exclude studies with 9 or fewer women. Minimum sample size is 10 women with recidivism information.
3. Women with only Category B sexual offences (Static-99R coding), such sex trade workers, or public indecency without an identifiable victim (sex in public places).
    a. Exception: Include women with sexual exploitation materials
    b. Exception: Include women prostituting children (for whatever motive).
4. Exclude studies that only included self-reported sexual offences or behaviours (e.g., anonymous surveys).
5. Exclude studies that simply report the proportion of women who have more than one offence. No separation between cohort collection period and follow-up time.

STUDY

Study Identification

Each study that met the above criteria is given a *study number*. If studies are based on an overlapping dataset, they are considered to be part of the same dataset. Consequently, each unique (non-overlapping) dataset was assigned a number (e.g., 8), and each study within the dataset assigned its own number following a decimal point (e.g., 8.1, 8.2).

Note if the study includes empirical analyses of recidivism risk factors for sexual recidivism YES/NO

Study Descriptive Information

For each dataset, only one Study Identification booklet will be completed. The Study Identification form will include study identifiers as well as study descriptive statistics. An example of the Study Identification booklet is included at the end of this manual. Study Identification and Description forms include the following variables.

Basic Study Descriptives

PUBLISHED

Whether the study is published or unpublished. When there are multiple studies both published and unpublished, coded this item based on the study from which most effect sizes were obtained. Journal articles (including *in press* articles), and book chapters are considered published sources. Manuscripts (unpublished or submitted), conference presentations, government reports, raw data, and dissertations are considered unpublished.

Any article or other source from which the recidivism rates cannot be coded from the information in publication and thus requires other sources (e.g., contacting the head researcher of that article or study), is considered unpublished.

PEERREVIEW

Whether the study is peer-reviewed. Includes peer-reviewed journal articles and doctoral dissertations. Excludes book chapters, manuscripts, conference presentations, government reports, and Masters theses. If the effect size is from raw data but a similar effect size is available in a peer-reviewed document, can code as peer reviewed. Example: there's a published effect size but the author provides updated or more detailed information.

ADDINFO

Was additional information obtained for the purposes of this meta-analysis? For example, raw data and other information provided directly by the researchers. Do not include new analyses or re-analyzes of existing data (e.g., calculating a mean from on a frequency table). If yes, add a brief description of the additional information on the Cover Sheet.

YEAR

The year the study was published/completed. If there are multiple studies, code the year of the study that provided the most findings; if the multiple studies have the same number of effect sizes, code the date of the most recent study.

COUNTRY

The country where the study originated. Add new countries as necessary.

PURPOSE

Code YES if the recidivism rates of women with a history of sexual offending was mentioned in the title or abstract of the document. Code NO if the data was extracted from a source that did

not have a title or abstract, except when unpublished recidivism information was specifically generated for the purpose of this meta-analysis (in which case, code YES).

Sample Information

SIZE

The largest sample size of any recidivism statistic.

LOCNTYPE

Whether the women were selected from the community or an institution. Use “mixed” if the proportions in the smaller location is greater than 5% or unknown.

SAMPLE

The sampling frame or how the sample was identified.

PRESELECTION
These are the categories used for Static-99R/Static-2002R recidivism studies. Routine/complete samples include everybody in a jurisdiction (State of Wisconsin) or setting (Red Wing Prison).

TREATMENT

Concerns sex crime specific treatment.

JUVENILE

Studies that include more than 10% juveniles are excluded.

AGEYRS
AGESD
LOWAGE
HIGHAGE

Age: mean, standard deviation, lowest and highest ages. If several different ages are reported, use the values from the largest sample with recidivism information.

MAIN.RACE
RACIAL

The proportion of the sample in the largest race/ethnicity group (a measure of racial/ethnic heterogeneity)

Recidivism Information

FIXTIME

Was the follow-up time the same for all women in the study (SD = 0)? If no, then variable.

Sexual Recidivism – includes all sexually motivated offences, and offences involving the sexual exploitation of children (e.g., prostituting children). Both contact and non-contact offences.

Violent Recidivism – includes all sexual recidivism along with nonsexual violent offences. Violent offences involve direct confrontation with victim: e.g., robbery, threatening, assault.

General (Any) Recidivism – includes all criminal behaviour (sexual, non-sexual violence, non-violent). If possible, exclude technical violations and violations of conditional release.

COVER SHEET Female Recidivism (2022)

STUDY NUMBER____

TITLE

____

AUTHOR(S)______

____

YEAR(S)____

SOURCE (E.G., JOURNAL)_________ _

____

____

OTHER ARTICLES USED?____

____

____

____

____

Additional Information?

NOTES____

____

____

____

Does the study examine sexual recidivism risk factors? YES/NO

Date: __________________

Coder: ___

BASIC STUDY DESCRIPTIVES

* STUDY - Identification # _____

* PUBLISHED - material is a published paper _____

0 = no

1 = yes

* PEERREVIEW – material is peer reviewed ________

0 = no

1 = yes

* ADDINFO – was additional information obtained to code effect size? _______

0 = no

1 = yes

* YEAR - date which study was published/released/completed ________

* COUNTRY - country where study originated _____

0 = Canada

1 = U.S.

2 = Netherlands

3 = Australia

4 = [add as necessary]

* PURPOSE – Was the main purpose of the study to examine the recidivism rates of women with a sexual offending history? ___

0 = no

1 = yes

SAMPLE INFORMATION

* SIZE - final sample size _____

SAMPLING FRAME NAME – setting offenders selected from (e.g., Correctional Service of Canada – Prairie Region)

____

* LOCNTYPE _____

1 = institution

2 = community

3 = combined

* SAMPLE ________

1 = Corrections (prison or community)

2 = Treatment in correctional setting (prison or community)

3 = Psychiatric or forensic mental health setting

4 = Administrative Database (e.g., sexual offender registry)

5 = Courts

6 = Other: Specify ___________________________________

* PRESELECTION ______

1 = routine/complete sample

2 = treatment sample

3 = preselected high risk/need (includes specialized high-intensity treatment)

4 = other non-routine/complete (include mixed samples)

* TREATMENT - did the offenders receive treatment? _____

0 = no, mostly untreated (approximately 75% or more)

1 = mixed (some did; some didn't)

2 = yes, mostly treated (approximately 75% or more)

3 = unknown

* JUVENILE – Any juvenile sex offenders in the sample? ______

0 = No (adults only)

1 = Some juveniles included: approximate % ________________________

* AGEYRS - mean age of sample in years (1 decimal point) _____

* LOWAGE- Lowest age ____

* HIGHAGE- Highest age ____

* MAIN.RACE - % of sample of majority race _____

* RACIAL - Majority race of the sample _____

0 = White

1 = Black

2 = Latina

3 = Indigenous peoples

4 = East-Asian

5 = Other

Recidivism Information

* COHORT.START – the earliest at-risk date for the cohort (year) __________

* COHORT.END – the latest at-risk date for the cohort (year) __________

* FOLLOWUP.END – the last year of recidivism information (year) ________

* FOLLOWUP.MIN – __________________(months)

* FOLLOWUP.MAX – _________________(months)

* FOLLOWUP.MEAN – ________________(months)

* Fixed or variable follow-up time (FIXTIME) 1 = fixed; 2 = variable; 3 = no info ______

* Sources of recidivism information specified? (RECINFO) 1=yes; 2=no _________

* Number of recidivism sources used? (N.RECINFO) ________

* Check off all recidivism sources used:

National criminal records search (NATIONAL) __________

Local criminal records search (LOCAL) __________ (municipal, state, provincial)

Other (REC.OTHER) __________ (collateral contacts, unofficial reports from agencies)

* Minimum involvement in CJS that would qualify as recidivism (RCD.CRIT) ___________

0 = Arrests

1 = Charges (includes conviction)

1 = Convictions (excludes arrests not resulting in convictions)

2 = Return to Custody

3 = Unknown

* Was street time used to measure recidivism (STREET.TIME)? _____________

0 = no

1 = yes

* ATTRITION - % of initial cohort sample missing recidivism data ____________

Recidivism Coding Study ID: ________________

Page # ______________________________ TABLE: ________________________________

Type of Recidivism	%<br>nn.n	N<br>(recidivists)	N Total	Average Follow-up<br>(nn months)
**Sexual**				
Overall				
0 – 5 years				60 months
5 – 10 years				60 months
**Violent**				
Overall				
0 – 5 years				60 months
5 – 10 years				60 months
**General (Any Crime)**				
Overall				
0 – 5 years				60 months
5 – 10 years				60 months
