# Supplementary material for: The Sexual Recidivism Rates of Women Are Still Low: An Updated Meta‐Analysis
Source: Crim Behav Ment Health. 2025 Oct 13;36(2):53–62. doi: 10.1002/cbm.70014 (PMC13063210; doi:10.1002/cbm.70014)
Supplement: Supplementary file 2 — Supporting Information S2 [file CBM-36-53-s001.docx]

**Method**

**Search Strategy**

The following databases were searched between February 19^th^ and October 21^st^, 2024: Google Scholar, PsycINFO, EBSCOhost Web, and ProQuest. For each of the databases the following search terms were used: “women”, “sex offenders”, “female”, “sexual offense”, “sexual offending”, “sexual recidivism”, “recidivism”, “reoffending”, and “re-offending”. Duplicates were removed and reviews were thoroughly analyzed for additional studies that might have been missed. The conference programs of the American Society of Criminology (ASC), the International Association for the Treatment of Sexual Offenders (IATSO), and the Association for the Treatment and Prevention of Sexual Abuse (ATSA) were also searched for any topics relating to females and sexual offending. ASC and IATSO databases had searchers performed from 2010 to the present. ATSA only had programs from 2017 to the present as they performed a recent database system change, and the older files did not migrate properly. The presenters from these conferences with related topics were then contacted to see if their content contained any data on female sexual offenders and recidivism.

**Supplemental Materials**

**Search terms, dates and locations table**

| Database/Website | Date searched | Key words |
| --- | --- | --- |
| Google Scholar |  |  |
|  | February 19, March 1, 2024 | "female sexual offenders" AND "sexual recidivism" |
|  | March 4, October 21, 2024 | “women sex offen*” AND “recidivism” OR “reoffending” |
| PsycINFO |  |  |
|  | February 21, 2024 | "female sexual offenders" AND "recidivism" |
|  |  |  |
|  | March 1, October 21, 2024 | “female sex offen*” OR “women sex offen*” AND “recidivism” OR “reoffending” |
|  |  |  |
| EBSCOhost Web |  |  |
|  | February 23, October 21, 2024 | “female sex offen*” OR “women sex offen*” AND “recidivism” OR “reoffending” OR “re-offending” |
| ProQuest |  |  |
|  | March 2, October 21, 2024 | “female sex offen*” OR “women sex offen*” AND “recidivism” OR “reoffending” |
| International Association for the Treatment of Sexual Offenders IATSO | March 2, 2024 | “female” OR “women” |
|  |  |  |
| American Society of Criminology ASC | March 3, 2024 | “female” OR “women” |
|  |  |  |
| Association for the Treatment and Prevention of Sexual Abuse ATSA | March 29, 2024 | “female” OR “women” |
